# Multiple environmental stressors confine the ecological niche of the rotifer *Cephalodella acidophila*

**DOI:** 10.1111/fwb.12104

**Published:** 2013-05

**Authors:** Thomas Weisse, Nicole Laufenstein, Guntram Weithoff

**Affiliations:** * Research Institute for Limnology of the University of InnsbruckMondsee, Austria; † Institute for Limnology of the Austrian Academy of SciencesMondsee, Austria; ‡ Department of Ecology and Ecosystem Modelling, University of PotsdamPotsdam, Germany

**Keywords:** acid lakes, *Cephalodella acidophila*, life-table experiments, pH, rotifers

## Abstract

The planktonic food web in extremely acidic mining lakes is restricted to a few species that are either acidophilic or acidotolerant. Common metazoans inhabiting acidic mining lakes with a pH below 3 include rotifers in the genera *Cephalodella* and *Elosa*.

The life history response of *Cephalodella acidophila* to three environmental key factors, pH (2, 3.5, 5.0 and 7.0), temperature (10, 17.5 and 25 °C) and food concentration (10 000, 35 000 and 50 000 algal cells per mL), was investigated in a full factorial design using life-table experiments.

The effect of each of the three environmental variables investigated on the rotifer life cycle parameters (life span, fecundity and population growth rate) differed. *C. acidophila* is a stenoecious species with a pH optimum in the range 3–4 and a comparably high food threshold. Combining the laboratory results with field data, we conclude that *C. acidophila* is severely growth limited in its natural habitat. However, low pH alone is not harmful as long as temperatures are moderate to warm and food is abundant.

The population of *C. acidophila* in the field is maintained mainly due to release from competitors and predators.

## Introduction

Rotifers are widespread in virtually all aquatic habitats and play an important role in the pelagic food web. As is the case with ciliates, most of the more than 2000 rotifer species are suspension feeders, grazing upon bacteria, small algae and heterotrophic protists and serve as important food for higher trophic levels (Gliwicz, 2003).

The genus *Cephalodella* (Monogononta: Notommatidae) is among the most species-rich genera of the phylum Rotifera, with approximately 190 described species (Segers, 2007). This genus is characterised by great phenotypic similarity, rendering taxonomic identification difficult (Nogrady & Pourriot, 1995). As a consequence, our understanding of the distribution and ecology of many species is blurred by misidentifications and doubtful records, and new species are to be expected even from well-explored regions (Jersabek, Weithoff & Weisse, 2011). Jersabek *et al.* (2011) recently described the new species *Cephalodella acidophila* Jersabek, Weithoff & Weisse from highly acidic mining lakes (pH < 3) in East Germany (Lake 130) and northern Austria (Lake Langau). This species occurs in man-made habitats at low to moderate abundance (Weithoff *et al.*, 2010; Moser & Weisse, 2011b). In the Lake Langau, the population density ranged from five individuals per litre in summer to 22 individuals per litre in autumn; the peak was recorded when temperature was below 10 °C (Moser & Weisse, 2011b). Phytoplankton biomass in the mixolimnion of this lake is relatively constant, with seasonal variations ranging from 0.17 to 0.36 mg C L^−1^ (Moser & Weisse, 2011b); higher phytoplankton biomass was recorded in the monimolimnion of L. Langau.

As is the case for natural volcano lakes, man-made acidic mining lakes are extreme aquatic habitats characterised by iron, sulphate and heavy metal concentrations several orders of magnitude higher than in circumneutral lakes (summarised in Geller, Klapper & Salomons, 1998). Accordingly, biodiversity in these lakes is greatly reduced, and rotifers may represent the dominant or even sole metazoan taxon in the simplified planktonic food web (Gaedke & Kamjunke, 2006; Weithoff *et al.*, 2010; Moser & Weisse, 2011b). The present investigation is part of a larger project investigating whether the pelagial of acidic mining lakes is primarily colonised by pH specialists or generalists (Moser & Weisse, 2011a, c; Weisse *et al.*, 2011) and to determine what factors control these plankton communities. Secondly, we used acidic mining lakes as a suitable ecosystem model to test for the significance of strong habitat selection (Weisse *et al.*, 2011).

General aspects of the ecology of *Cephalodella* sp. (a closely related strain to *C. acidophila*) such as growth, reproduction and feeding have been investigated with a strain isolated from Lusatia, East Germany (Kamjunke *et al.*, 2004; Weithoff, 2004, 2005, 2007; Weithoff & Wacker, 2007). The strain used in these studies was an as yet unidentified species that was originally misidentified as *C. hoodi*.

The response of the rotifer community to decreasing pH has been studied mainly in moderately acidified (pH 4–7) North American lakes (Yan & Geiling, 1985; Brett, 1989; Frost *et al.*, 1998). From these studies and similar investigations in Sweden (Berzins & Pejler, 1987), it is known that the occurrence and abundance of rotifers in lakes are confined by pH (Wallace *et al.*, 2006; Segers, 2007). The distribution of rotifers in highly acidic (pH < 3) lakes has been investigated in Lusatia, East Germany (reviewed by Deneke, 2000). The effect of pH on various life history parameters has been studied experimentally with five *Brachionus* species over the pH range 5–10 (Yin & Niu, 2008). An experimental investigation on the pH response of rotifers at lower pH is, to our knowledge, still lacking.

We used a *C. acidophila* clone isolated from a small acid mining lake at Langau, Austria, to investigate whether this species is an acidophil (i.e. specifically adapted to the harsh environmental conditions prevailing in acid mining lakes). Alternatively, *C. acidophila* may be acidotolerant, taking refuge from competitors or predators that are more dominant in circumneutral or weakly acidified lakes. Since the investigations cited earlier revealed that seasonal temperature variation in these small lakes may range from 0 to 30 °C and food is generally scarce, we investigated the interactive effect of pH, temperature and food level on the fitness of the rotifers. Our hypothesis was that the combined stress effect of low pH, extreme temperature and limiting food supply would confine the ecological niche of *C. acidophila* to a relatively narrow habitat range compared to more common rotifers. The significance of the interactive effect of the two abiotic factors for the width of the realised niche width has already been demonstrated for the dominant flagellate species in these acidic mining lakes (Moser & Weisse, 2011a). We used life-table experiments to monitor the survival, reproduction and hatching success of the rotifers.

## Methods

### Isolation of the rotifer strain and stock cultures

The food alga, *Chlamydomonas acidophila* Negoro, and the rotifer *Cephalodella acidophila* were isolated from an acidic mining lake located at Langau (Lower Austria, 48°50′N, 15°43′E). Algal stock cultures were maintained as batch cultures in 50-mL culture tissue flasks with modified Woods Hole Medium (MWC) at a continuous light intensity of 90–100 mmol m^−2^ s^−1^ and 17.5 °C. pH ranged from 2.8 to 3.0 to mimic the natural situation (Moser & Weisse, 2011b). Rotifers were kept in identical flasks and the same medium with *Chlamydomonas* as food, but at 15 °C. From these raw stock cultures, a small volume was poured into Petri dishes; individual females were selected and transferred with a pipette to 12-well tissue plates (one female per well). If eggs were produced, they were transferred individually to the next well. This procedure was repeated five times to obtain clonal rotifer cultures. In the final step, we waited until several individuals had hatched. These were then transferred to 50-mL culture flasks and yielded the clonal stock culture. All experiments were performed with the same clone that had reached the largest population size over a period of 3 weeks of continuous culture.

### Experimental culture conditions – life-table experiments

Subcultures of the algae and rotifers (100 mL volume each) were gradually acclimated to the respective experimental conditions using a similar procedure as applied earlier in our laboratory with protists (Weisse & Stadler, 2006; Moser & Weisse, 2011a). The longest adaptation period (∼2 weeks) was used for the lowest temperature and pH investigated. The pH was measured daily with a pH meter (Seven Easy pH Meter S20; Mettler Toledo, Vienna, Austria) to the nearest 0.01 unit. If pH deviated by more than 0.2 units from the target value, it was adjusted by the addition of small amounts of 0.1 or 1 m NaOH or HCl, respectively. The algal concentrations were monitored in each treatment using an electronic particle counter (CASY 1-Model TTC; Schärfe System, Reutlingen, Germany; Weisse & Kirchhoff, 1997).

From these acclimatised cultures, rotifer eggs were collected and transferred to Petri dishes with sterile medium. Next, at least 50 eggs per treatment were pipetted into wells of 6-well microtitre plates with the medium and food algae adjusted to the respective experimental conditions and incubated overnight. The next morning, at the beginning of the experiments, neonates were individually transferred to 24 wells of a 96-well microtitre plate filled with 200 μL of acclimated algae in MWC at the respective experimental conditions. Experiments were performed in the dark to prevent the food algae from growing. Twenty-four animals were used in each treatment. The surviving individuals were transferred daily into fresh wells of a microtitre plate filled with 200 μL of fresh medium and the target food concentration. The survival of the animals, number of eggs produced and their viability were individually recorded until all initial animals had died. We never found resting eggs or males in our stock cultures (i.e. there was no hint of sexual reproduction in our study animals).

Experiments were conducted at three temperatures (10, 17.5 and 25 °C), three food levels (10 000 *Chlamydomonas* cells per mL, 35 000 *Chlamydomonas* cells per mL and 50 000 *Chlamydomonas* cells per mL) and four pH levels (2.65, 3.5, 5.0 and 7.0). Accordingly, the total experimental set consisted of 36 treatments. The food concentrations were chosen according to the abundance of *Chlamydomonas acidophila* encountered in the acid mining lake at Langau (Moser & Weisse, 2011b).

Cellular abundance was converted to carbon units, assuming 0.23 pg C μm^−3^ (Tittel *et al.*, 2005), and the average cell volume of *Chlamydomonas* was measured for the cultured cells at each temperature and pH (Laufenstein, 2010). Cells were larger at 10 °C, resulting in a carbon concentration of 0.29 mg L^−1^ at the lowest cellular abundance; cells were smaller at the higher temperatures, resulting in a carbon concentration of 0.16 mg L^−1^. Accordingly, the lowest food level was below the food threshold concentration (0.34 mg C L^−1^) determined for a Lusatian clone of *Cephalodella* sp. at pH = 2.65 and 20 °C (Weithoff, 2005). Thus, at the same resource density in terms of cell numbers at 10 °C, the animals had a higher resource availability than at higher temperatures in terms of carbon. However, all cell sizes were well within the range that can be ingested by *Cephalodella*. The maximum *Chlamydomonas* biomass in the experiments (1.71 mg C L^−1^) was reached at the highest cell number, pH 2.65 and 10 °C.

### Calculation of experimental results

The net reproductive rate *R*_0_ was calculated according to Birch (1948):


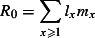
(1)

where *x* is the time in days, *m*_*x*_ is the number of viable eggs per surviving female at time *x*, and *l*_*x*_ is the proportion of surviving females at time *x*.

Instantaneous growth rates of the populations (*r*) were estimated from the survival and reproduction data of life-table experiments by solving the Euler–Lotka equation iteratively (Birch, 1948):


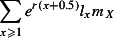
(2)

Equation 2 assumes that all births in an interval between two consecutive observations occur at the mid-point. The symbols have the same meaning as in the previous equation.

All graphs were drawn using SigmaPlot for Windows version 11.0 (Systat Software, Inc., San Jose, CA, U.S.A.). We created a 3D mesh plot to visualise reproduction (juveniles per female) of *C. acidophila* in relation to pH and food. The SigmaPlot curve fitter uses the Marquardt–Levenberg algorithm to find the coefficients (parameters) of the independent variables that give the best fit between the equation and the data. We did not interpolate or smooth our data.

### Statistical analyses

We investigated survival and fecundity of *Cephalodella acidophila* at four different pH, three temperatures and three food levels. Three-way anova and Tukey’s test were used to test for significant effects of each factor on life span, egg production and net reproductive rate of *C. acidophila*, and to determine which treatments differed significantly (*P* < 0.05). For the analysis of life span, total number of eggs and number of neonates each individual female was a replicate; for the hatching proportion, we used the proportion of the whole cohort as input variable. The statistical analyses were performed with SigmaStat for Windows version 2.03 (SPSS Inc., Chicago, IL, U.S.A.).

## Results

### The life span of *Cephalodella acidophila* in response to pH, temperature and food level

**Fig. 1:**
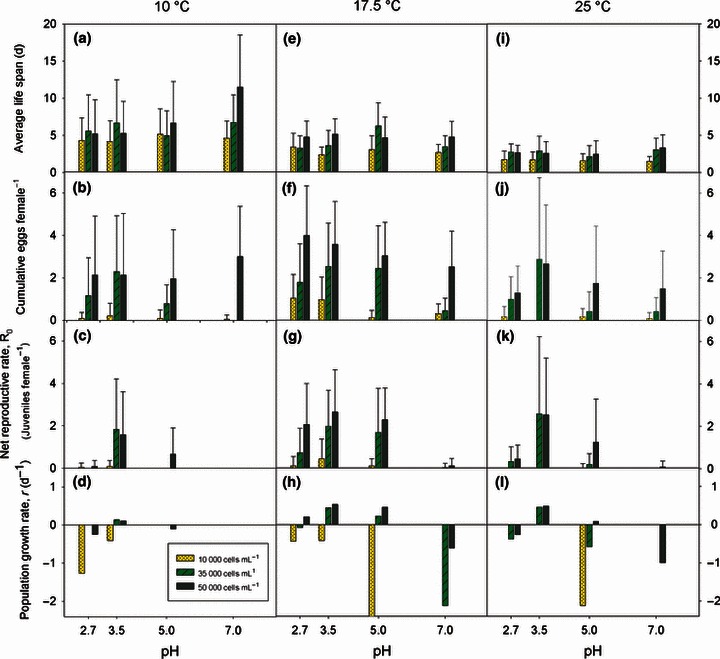
Life cycle parameters of *Cephalodella acidophila* in response to pH, temperature and food levels. (a, e, j). Average life span of females; (b, f, j) cumulative egg production per female; (c, g, k) net reproductive rate *R*_0_; (d, h, l) population growth rate *r*. Experimental temperature is indicated on top of each column. Bars denote standard errors.

The average life span of the rotifers (Fig. 1a,e,i) was significantly affected by temperature (*P* < 0.001) and food (*P* = 0.003), while the pH effect was not significant (*P* = 0.300) when considering the whole data set. Effects were most obvious at the intermediate temperature. At 17.5 °C, *C. acidophila* lived significantly longer (Tukey’s test) at the highest food level, compared to the lowest food concentration, at all pHs tested (Fig. 1e). Significant differences in the average life span of *C. acidophila* were also observed at the highest experimental temperature and pH 2.7 and 7.0 (Fig. 1i). There was no significant effect of food level at 10 °C (Fig. 1a).

The maximum age of an individual female (27 days) was recorded under a combination of low temperature, high pH and high food supply. At the lowest temperature tested (10 °C), the maximum life span under food-replete conditions exceeded 17 days in each experimental treatment, irrespective of pH (Fig. S1, see Supporting Information). At the highest temperature, the maximum life span was always <10 days.

### Environmental effects on reproduction of *Cephalodella acidophila*

Egg production of the females (Fig. 1b,f,j) was sensitive to all three environmental factors, being significantly affected by food level, temperature and pH (three-way anova, *P* < 0.001 in each case). Secondly, there was a significant interaction between all variables (food level × temperature × pH, *P* = 0.007); that is, the effect of each factor was affected by the other two. *Cephalodella acidophila* produced more eggs when more food was available. However, we found a discrepancy between egg production and net reproductive rate *R*_0_ that is explained by differential hatching success. The latter was primarily affected by pH (*P* < 0.001). Temperature (*P* = 0.026) and food (*P* = 0.042) also significantly affected hatching success. Thus, the net reproductive rate *R*_0_ (the total average number of juveniles produced per female; eqn 1) varied even more than egg production in response to the environmental factors (Fig. 1c,g,k). At the lowest experimental temperature tested, 10 °C, *R*_0_ exceeded 1, the theoretical minimum to sustain a population, only at pH 3.5 (Fig. 1c). The rotifers reached their highest reproductive success at 17.5 °C; if sufficient food was available, each female produced an average of more than two offspring at pHs ranging from 2.7 to 5.0 (Fig. 1g). This plot also illustrates that in contrast to acidic conditions, only a minority (*c*. 2%) of the eggs hatched and survived under neutral conditions. At the highest temperature investigated, *C. acidophila* produced more than one juvenile per female at pH 3.5–5.0, provided that food supply was sufficient (Fig. 1k).

The results of *R*_0_ translate to the population growth rate (*r*, eqn 2) of *C. acidophila* (Fig. 1d,h,l). At the intermediate temperature, the rotifer population can survive at pHs ranging from 2.7 to 5.0 under food-replete conditions (Fig. 1h). The width of the pH niche of the rotifers is narrowed both at lower (Fig. 1d) and at higher (Fig. 1l) temperatures.

**Fig. 2:**
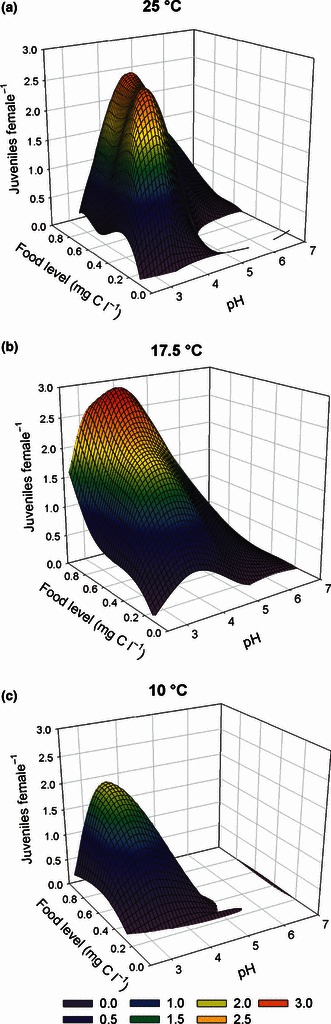
3D mesh plots of the net reproductive rate (juveniles female^−1^) of *Cephalodella acidophila* in response to pH, food level and temperature.

We used our experimental data to visualise the reproduction of *C. acidophila* over the entire pH range from 2.5 to 7.0 and food levels ranging from 0 to 1.0 mg C L^−1^ (Fig. 2). The 3D plots illustrate that net reproductive rate is primarily affected by food level and pH and shows a clear optimum close to pH 3.5. Temperature is less important than pH and food but affects the maximum number of offspring produced per female. Note that the net reproductive rate does not peak at the highest temperature, but at 17.5 °C (Fig. 2b). Figure 2 also suggests that *C. acidophila* needs a relatively high threshold food concentration of 0.4–0.6 mg C L^−1^ to sustain its population. Remarkably, this threshold food concentration was highest at the minimum experimental temperature tested.

## Discussion

### *Cephalodella acidophila*, a stenoecious, acidophil rotifer species

Our results corroborate earlier experimental studies with a closely related *Cephalodella* species isolated from Lake 111 in Lusatia, Germany (Weithoff, 2005, 2007; Weithoff & Wacker, 2007). Both strains, originating from similar habitats, exhibit similar life span, growth rates and threshold food concentrations.

The pH response was not studied in the previous investigations. Here, we investigated the combined (stress) effect of pH, temperature and food level on the fecundity of *Cephalodella acidophila*. To our knowledge, ours is the first study to investigate the combined effect of these key environmental variables on the life cycle of any planktonic metazoan. Thus far, similar work had been performed only for some protist species (Weisse *et al.*, 2002, 2007, 2012). Two major conclusions of the present study are similar to the earlier investigations with protozoa. First, summarising various experimental work with the small acidotolerant ciliate *Urotricha* spp., Weisse (2006) reported that under the seasonal variation typical of a mesotrophic, circumneutral lake, temperature had the strongest effect on the fitness of the ciliates, followed by food supply and pH. Weisse & Stadler (2006) inferred that the pH effect on growth rates appears to be minor in a given water body at circumneutral pH, but may restrict the occurrence and distribution of freshwater ciliates in contrasting environments. Virtually the same conclusion applies for the rotifer *C. acidophila*, which is confined to acid lakes. Seasonal variation of pH in the epilimnion of the acid mining lake at Langau rarely exceeds one pH unit (Moser & Weisse, 2011b). Although pH is measured on a logarithmic scale, the effect of the seasonal pH variation is of minor importance relative to the large temperature fluctuations (between 0 and 30 °C, Moser & Weisse, 2011b) and more than 10-fold seasonal variation in *Chlamydomonas acidophila* biomass recorded in acidic mining lakes (Weithoff *et al.*, 2010). Secondly, as recently demonstrated for freshwater protists (Moser & Weisse, 2011a), the width of the pH niche of the rotifers in acidic mining lakes is significantly affected by temperature and food supply. For example, the population growth rate (*r*) measured at 10 °C, pH of 3.5 and high food levels is positive and similar to *r* measured at 25 °C, high food level but at the suboptimal pH of 5.0. If the pH response is investigated under optimised laboratory conditions (i.e. close to the temperature optimum and under food-replete conditions), the fundamental pH niche of the species under investigation may be assessed. This has, however, little meaning in the field if temperature and food supply become unfavourable or limiting. Using a realistic range of the environmental key variables, we demonstrated that the realised pH niche of *C. acidophila* may be narrowed by two pH units, and confined to little more than one pH unit around pH 3.5, relative to its near-to-fundamental pH niche. If the survival of the rotifer population is assessed across aquatic habitats of widely differing pH, food and pH are the primary factors limiting the rotifer reproduction rate.

Our experimental data suggest that the bottleneck in the survival of the rotifer population is reached in winter, when low *in situ* food levels coincide with a high food threshold of the rotifers (cf. Fig. 2c). We classify *C. acidophila* as a specialised, stenoecious species, with a temperature optimum close to 17 °C and a pH optimum close to 3.5. Experiments with Lusatian rotifer strains had shown that *Cephalodella* sp. had a lower optimum temperature for growth than the sympatric species *Elosa worallii* Lord (Weithoff, 2004).

It is important to note that the facultatively parthenogenetic, monogonont rotifers can endure unfavourable environmental conditions by producing resting eggs, thus bypassing environmental bottlenecks. Whether this applies to *C. acidophila* remains an open question; neither resting eggs nor males have been described for this species thus far or have been found in our stock cultures. Since sampling frequency has usually been low (mostly monthly sampling intervals) in the acidic mining lakes investigated, a switch in the life cycle of *Cephalodella* to sexual reproduction and the production of resting eggs might have been missed. Mixis in other monogonont rotifer genera such as *Brachionus* and *Synchaeta* is typically induced chemically via crowding (Gilbert, 1963; Timmermeyer & Stelzer, 2006). For *C. acidophila*, this is unlikely in the field because population densities are low (Weithoff *et al.*, 2010; Moser & Weisse, 2011b) so that a crowding effect might not occur; even if females become mictic, there would only be a very low chance that a male would find a mating partner.

### Differential sensitivity of survival and fecundity to environmental variables

We used life-table experiments to differentiate between the effects of environmental key variables on the vitality of the adult females and of their eggs. The statistical analyses revealed that temperature is the master factor affecting the life span of the female rotifers. At low temperature and moderate food supply, single *C. acidophila* females may survive more than 2 weeks, irrespective of pH. We infer that if single *C. acidophila* females are dispersed to circumneutral lakes, they may feed and produce eggs, but since the hatching success of the eggs is close to zero, the rotifer cannot establish in the new environment.

Egg production is primarily sensitive to food supply (e.g. Weithoff, 2007), but a high egg production rate does not necessarily translate to a high population growth rate. All three factors (pH, food supply and temperature) together account for the net reproduction rate (*R*_0_) of the species. In the field, *R*_0_ must exceeded 1, the theoretical minimum to sustain a population, to compensate for losses due to predation and parasitism, which usually can be ignored in laboratory experiments.

In the laboratory, *C. acidophila* from a Lusatian lake (L 129) and the closely related *Cephalodella* sp. may reach a maximum population growth rate (*r*_max_, intrinsic growth rate) of 0.68 day^−1^ at 20 °C (Weithoff, 2005), which is, as the authors pointed out, higher than *r*_max_ of most planktonic rotifer species under comparable resource conditions. However, the food at *r*_max_ was well above values found in nature (Weithoff, 2005; Weithoff *et al.*, 2010; Moser & Weisse, 2011b).

### Implications for the field and conclusions

Our laboratory results demonstrate that *C. acidophila* is obligately acidophilic (i.e. it cannot survive at neutral pH). Based on our findings, *C. acidophila* achieves positive population growth rates only in a very restricted range of environmental conditions (pH, temperature and food concentration). Field data from the Lake Langau, the lake the present strain was isolated from, indicate that conditions for most of the time are distinctly below optimal, suggesting that the realised growth rates of *Cephalodella* in the field are low (Weithoff *et al.*, 2010). This is in accordance with results from rotifers from the Lusatian region where resource limitation lasted throughout the whole vegetation period, with *Cephalodella* sp. being more severely resource-limited than the sympatric *Elosa worallii* (Weithoff, 2004). The only way to maintain a population under conditions far from optimal is by keeping loss rates low. No rotiferan competitors such as *Elosa worallii* have been found in the Lake Langau. Moreover, the only potential predator of rotifers at pHs < 3, the heliozoan species *Actinophrys sol* (Bell, Weithoff & Gaedke, 2006), has not been detected in the Lake Langau.

In conclusion, *C. acidophila* is bottom-up regulated in the Lake Langau and benefits from release from competitors and predators. As for other taxa with narrow environmental niches (Williams *et al.*, 2009), the small geographic range of *C. acidophila* seems to be associated with low local abundance.

## Supporting information

**Figure S1.** Cumulative survival of the femaleCephalodella *Cephalodella acidophila* at low (open symbols), intermediate (grey filled symbols) and high (black filled symbols) food supply at three experimental temperatures and four different pHs.

## References

[b1] Bell EM, Weithoff G, Gaedke U (2006). Temporal dynamics and growth of *Actinophrys sol* (Sarcodina: Heliozoa), the top predator in an extremely acidic lake. Freshwater Biology.

[b2] Berzins B, Pejler B (1987). Rotifer occurrence in relation to pH. Hydrobiologia.

[b3] Birch LC (1948). The intrinsic rate of natural increase of an insect population. Journal of Animal Ecology.

[b4] Brett MT (1989). The rotifer communities of acid-stressed lakes of Maine. Hydrobiologia.

[b5] Deneke R (2000). Review of rotifers and crustaceans in highly acidic environments of pH values <3. Hydrobiologia.

[b6] Frost TM, Montz PK, Gonzalez MJ, Sanderson BL, Arnott SE (1998). Rotifer responses to increased acidity: long-term patterns during the experimental manipulation of Little Rock Lake. Hydrobiologia.

[b7] Gaedke U, Kamjunke N (2006). Structural and functional properties of low- and high-diversity planktonic food webs. Journal of Plankton Research.

[b8] Geller W, Klapper H, Salomons W (1998). Acidic Mining Lakes: Acid Mine Drainage, Limnology and Reclamation.

[b9] Gilbert JJ (1963). Mictic female production in the rotifer *Brachionus calyciflorus*. Journal of Experimental Zoology.

[b10] Gliwicz ZM, O’sullivan P, Reynolds CS (2003). Zooplankton. The Lakes Handbook.

[b12] Jersabek CD, Weithoff G, Weisse T (2011). *Cephalodella acidophila* n. sp. (Monogononta: Notommatidae), a new rotifer species from highly acidic mining lakes. Zootaxa.

[b13] Kamjunke N, Gaedke U, Tittel J, Weithoff G, Bell EM (2004). Strong vertical differences in the plankton composition of an extremely acidic lake. Archiv für Hydrobiologie.

[b14] Laufenstein N (2010). Lebenszyklus einer acidophilen *Cephalodella*-Art in Abhängigkeit von verschiedenen Futterkonzentrationen, pH-Werten und Temperaturen.

[b15] Moser M, Weisse T (2011a). Combined stress effect of pH and temperature narrows the niche width of flagellates in acid mining lakes. Journal of Plankton Research.

[b16] Moser M, Weisse T (2011b). The most acidified Austrian lake in comparison to a neutralized mining lake. Limnologica.

[b17] Moser M, Weisse T (2011c). The outcome of competition between the two chrysomonads *Ochromonas* sp. and *Poterioochromonas malhamensis* depends on pH. European Journal of Protistology.

[b18] Nogrady T, Pourriot R, Segers H (1995). Rotifera, Vol. 3: The Notommatidae and The Scaridiidae.

[b19] Segers H (2007). Annotated checklist of the rotifers (Phylum Rotifera), with notes on nomenclature, taxonomy and distribution. Zootaxa.

[b20] Timmermeyer N, Stelzer C-P (2006). Chemical induction of mixis in the rotifer *Synchaeta tremula*. Journal of Plankton Research.

[b21] Tittel J, Bissinger V, Gaedke U, Kamjunke N (2005). Inorganic carbon limitation and mixotrophic growth in *Chlamydomonas* from an acidic mining lake. Protist.

[b22] Wallace RL, Snell TW, Ricci C, Nogrady T (2006). Rotifera 1: Biology, Ecology and Systematics.

[b23] Weisse T (2006). Freshwater ciliates as ecophysiological model organisms – lessons from *Daphnia*, major achievements, and future perspectives. Archiv für Hydrobiologie.

[b24] Weisse T, Berendonk T, Kamjunke N, Moser M, Scheffel U, Stadler P (2011). Significant habitat effects influence protist fitness: evidence for local adaptation from acidic mining lakes. Ecosphere.

[b25] Weisse T, Kirchhoff B (1997). Feeding of the heterotrophic freshwater dinoflagellate *Peridiniopsis berolinense* on cryptophytes: analysis by flow cytometry and electronic particle counting. Aquatic Microbial Ecology.

[b26] Weisse T, Moser M, Scheffel U, Stadler P, Berendonk T, Weithoff G (2012). Systematics and species-specific response to pH of *Oxytricha acidotolerans* sp. nov. and *Urosomoida* sp. (Ciliophora, Hypotricha) from acid mining lakes. European Journal of Protistology.

[b27] Weisse T, Scheffel U, Stadler P, Foissner W (2007). Local adaptation among geographically distant clones of the cosmopolitan freshwater ciliate *Meseres corlissi* II. Response to pH. Aquatic Microbial Ecology.

[b28] Weisse T, Stadler P (2006). Effect of pH on growth, cell volume, and production of freshwater ciliates, and implications for their distribution. Limnology and Oceanography.

[b29] Weisse T, Stadler P, Lindström ES, Kimmance SA, Montagnes DJS (2002). Interactive effect of temperature and food concentration on growth rate: a test case using the small freshwater ciliate *Urotricha farcta*. Limnology and Oceanography.

[b30] Weithoff G (2004). Vertical niche separation of two consumers (Rotatoria) in an extreme habitat. Oecologia.

[b31] Weithoff G (2005). On the ecology of the rotifer *Cephalodella hoodi* from an extremely acidic lake. Freshwater Biology.

[b32] Weithoff G (2007). Dietary restriction in rotifers – the effect of the length of food deprivation on life span and reproduction. Oecologia.

[b33] Weithoff G, Moser M, Kamjunke N, Gaedke U, Weisse T (2010). Lake morphometry strongly shapes the plankton community structure in acidic mining lakes. Limnologica.

[b34] Weithoff G, Wacker A (2007). The mode of nutrition of mixotrophic flagellates determines the food quality for their consumers. Functional Ecology.

[b35] Williams SE, Williams YM, Vanderwal J, Isaac JL, Shoo LP, Johnson CN (2009). Ecological specialization and population size in a biodiversity hotspot: how rare species avoid extinction. Proceedings of the National Academy of Sciences.

[b36] Yan ND, Geiling W (1985). Elevated planktonic rotifer biomass in acidified metal-contaminated lakes near Sudbury, Ontario. Hydrobiologia.

[b37] Yin XW, Niu CJ (2008). Effect of pH on survival, reproduction, egg viability and growth rate of five closely related rotifer species. Aquatic Ecology.

