# Supplementary figures and images for: Multiple environmental stressors confine the ecological niche of the rotifer Cephalodella acidophila
Source: Freshw Biol. 2013 May;58(5):1008–15. doi: 10.1111/fwb.12104 (PMC3659023; doi:10.1111/fwb.12104)

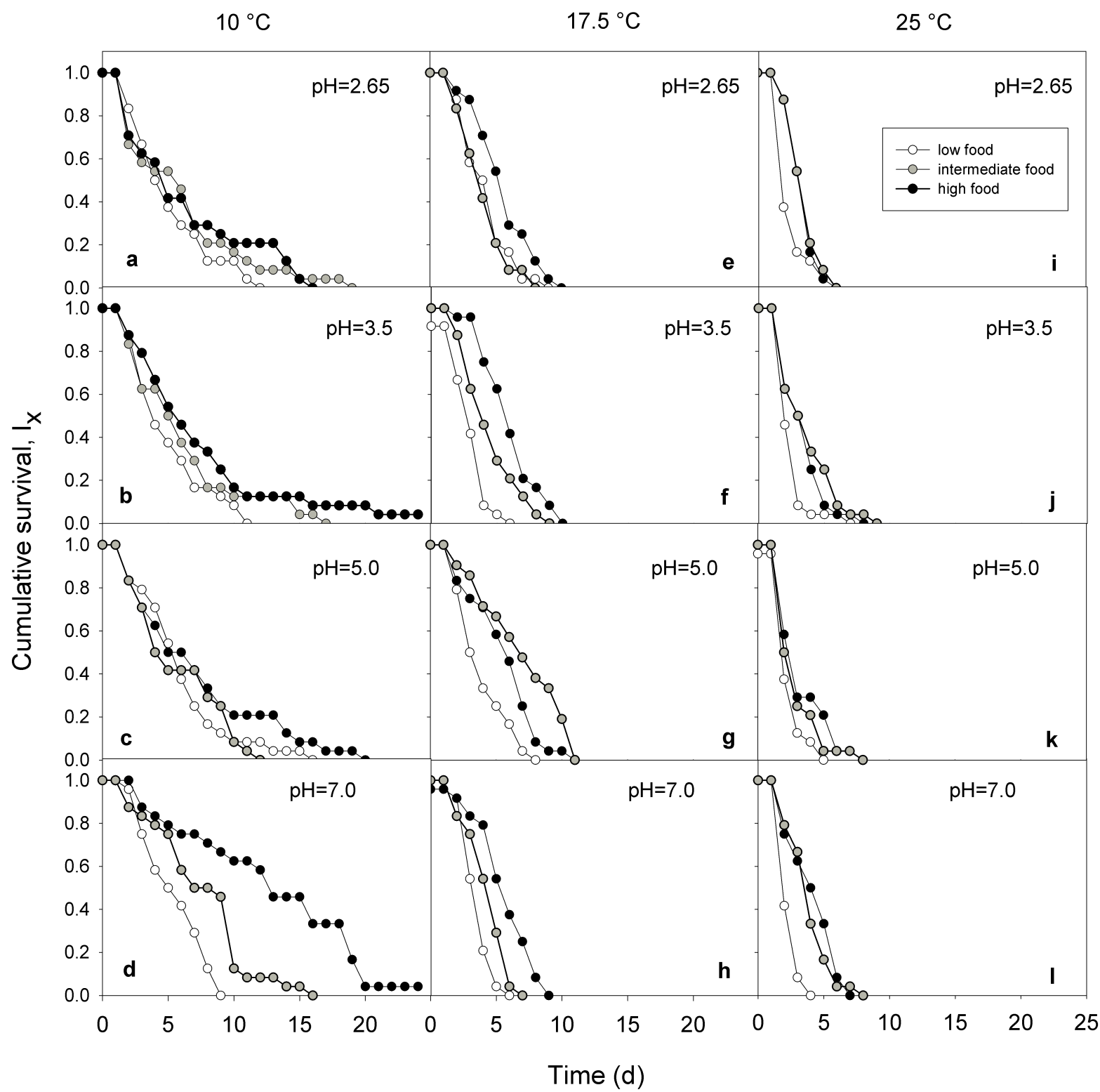

Supplement: Supplementary file 1 [file fwb0058-1008-SD1.pdf]
